# Meet the Section Editor

**DOI:** 10.2174/1570159X2110230626141250

**Published:** 2023-08-15

**Authors:** Gianluca Rosso

**Affiliations:** 1 Department of Neurosciences ‘Rita Levi Montalcini, University of Turin, Torino, Italy

Associate Professor of Psychiatry at Department of Neurosciences ‘Rita Levi Montalcini’, University of Torino, Italy. Clinical activity at Psychiatric Unit of San Luigi Gonzaga University Hospital of Orbassano (Torino), Italy. Lecturer at University of Turin: Medicine and Surgery School and other Degree Courses, Postgraduate School of Psychiatry. Author of over 100 publications on national and international peer reviewed journals. Research interests: mood disorders, clinical psychopharmacology, obsessive-compulsive disorder, short-term psychodynamic psychotherapy.
